# Sulforaphane Restores Mitochondrial β-Oxidation and Reduces Renal Lipid Accumulation in a Model of Releasing Unilateral Ureteral Obstruction

**DOI:** 10.3390/antiox14030288

**Published:** 2025-02-28

**Authors:** Ana Karina Aranda-Rivera, Isabel Amador-Martínez, Omar Emiliano Aparicio-Trejo, Juan Carlos León-Contreras, Rogelio Hernández-Pando, Emma Saavedra, Fernando E. García-Arroyo, José Pedraza-Chaverri, Laura Gabriela Sánchez-Lozada, Edilia Tapia

**Affiliations:** 1 Departamento de Biología, Facultad de Química, Universidad Nacional Autónoma de México, Ciudad Universitaria, Coyoacán, Mexico City 04510, Mexico; 2 Posgrado en Ciencias Biológicas, Universidad Nacional Autónoma de México, Ciudad Universitaria, Coyocán, Mexico City 04510, Mexico; 3 Departamento de Fisiopatología Cardio-Renal, Instituto Nacional de Cardiología Ignacio Chávez, Mexico City 14080, Mexico; 4 Departamento de Patología, Instituto Nacional de Ciencias Médicas y Nutrición “Salvador Zubirán”, Mexico City 14080, Mexico; 5 Departamento de Bioquímica, Instituto Nacional de Cardiología “Ignacio Chávez”, Mexico City 14080, Mexico

**Keywords:** obstructive nephropathy (ON), sulforaphane (SFN), mitochondrial biogenesis, β-oxidation, fatty acid metabolism, mitochondrial dynamics

## Abstract

Obstructive nephropathy (ON), characterized by urine flow disruption, can induce chronic kidney disease (CKD). Although the release of the obstruction is performed as the primary intervention, renal pathology often persists and progresses. Accordingly, the murine model of releasing unilateral ureteral obstruction (RUUO) is valuable for investigating the molecular events underlying renal damage after obstruction release. Remarkably, after RUUO, disturbances such as oxidative stress, inflammation, lipid accumulation, and fibrosis continue to increase. Mitochondrial dysfunction contributes to fibrosis in the UUO model, but its role in RUUO remains unclear. Additionally, the impact of using antioxidants to restore mitochondrial function and prevent renal fibrosis in RUUO has not been determined. This study aimed to determine the therapeutic effect of pre-administering the antioxidant sulforaphane (SFN) in the RUUO model. SFN was administered 1 day before RUUO to evaluate mitochondrial biogenesis, fatty acids (FA) metabolism, bioenergetics, dynamics, and mitophagy/autophagy mechanisms in the kidney. Our data demonstrated that SFN enhanced mitochondrial biogenesis and reestablished mitochondrial oxygen consumption and β-oxidation. These effects collectively reduced lipid accumulation and normalized mitochondrial dynamics, mitophagy, and autophagy, thereby mitigating fibrosis after obstruction. Our findings suggest that SFN holds promise as a potential therapeutic agent in ON-induced CKD progression in RUUO and opens new avenues in studying antioxidant molecules to treat this disease.

## 1. Introduction

Obstructive nephropathy (ON) globally affects neonates and adults and is caused by urinary tract diseases such as renal calculi, prostatic hyperplasia, bladder tumors, and congenital anatomical abnormalities [1,2]. In this condition, the urine flow is interrupted, causing hemodynamic alterations and changes in glomerular filtration and kidney architecture, leading to chronic kidney disease (CKD) [3,4]. CKD affects about 11% of the global population and is one of the leading causes of death worldwide [5]. Although in ON, obstruction relief surgery is the primary treatment strategy, it has been suggested that renal damage continues even after the obstruction is removed [6,7,8]. The release of the unilateral ureteral obstruction (RUUO) model has been implemented to investigate the pathological mechanisms following the obstruction removal [6,9]. Previously, we and other authors reported the persistence of oxidative stress, inflammation, and apoptosis in the kidney after unilateral ureteral obstruction (UUO) removal, which even intensified, producing increased renal damage [8,10,11]. Specifically, we demonstrated that even with the satisfactory release of UUO, oxidative damage persisted, and the activity of antioxidant enzymes was not carried out correctly. Moreover, inflammatory and apoptotic mechanisms also continued and increased [10], indicating that RUUO by itself could not alleviate renal damage originated by UUO. Therefore, alternative strategies must be addressed to reverse the functional and pathological changes in RUUO.

Mitochondrial dysfunction is critical in fibrosis development in the UUO model [12,13]. For example, distinct processes such as reduced mitochondrial biogenesis, altered mitochondrial dynamics and mitophagy, and metabolic reprogramming promote the development of fibrosis in UUO [12,14,15]. However, if these alterations continue after the RUUO is poorly understood. Additionally, no treatments focused on mitochondrial dysfunction in the RUUO model have been reported.

Sulforaphane (SFN) is an antioxidant found in cruciferous vegetables like broccoli and sprouts. It promotes mitochondrial biogenesis in the UUO model, which restores lipid metabolism, mitochondrial dynamics, and mitophagy, preventing kidney damage and fibrosis [16,17]. Furthermore, in the RUUO model, SFN can activate the nuclear factor erythroid 2-related factor 2 (Nrf2), consequently decreasing oxidative stress, inflammation, and apoptosis [10]. Notably, SFN restored S-glutathionylation in mitochondria, suggesting that SFN had a protective effect on these organelles [10]. However, whether SFN might prevent fibrosis progression after RUUO by targeting mitochondria is unknown.

In the current study, we administered SFN before removing UUO to evaluate mitochondrial biogenesis, fatty acids (FA) metabolism, bioenergetics, dynamics, and mitophagy/autophagy mechanisms in the kidney.

## 2. Materials and Methods

### 2.1. Reagents and Chemicals

**Figure 1:**
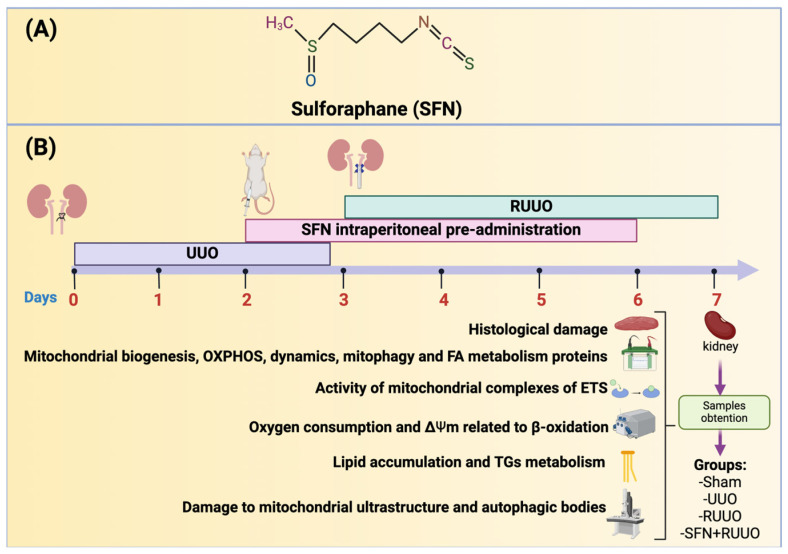
Sulforaphane (SFN) structure and experimental strategy. (**A**) SFN chemical structure. (**B**) Unilateral ureteral obstruction (UUO) surgery was carried out on day 0 and maintained for three days. SFN administration began two days after UUO (day 2 in the figure) and continued until day six. On day three, the release of UUO (RUUO) was performed. Finally, samples were obtained on day seven. Kidney samples were used to determine histological damage, protein levels related to mitochondrial biogenesis, dynamics, oxidative phosphorylation (OXPHOS), mitophagy, and fatty acid (FA) metabolism. Moreover, the activity of mitochondrial complexes of the electron transfer system (ETS), oxygen consumption by high-resolution respirometry, and mitochondrial membrane potential (ΔΨm) related to β-oxidation were evaluated in isolated mitochondria. We also determined lipid accumulation and triglycerides (TGs) metabolism and the damage to mitochondrial ultrastructure and content of autophagic bodies.

R,S-SFN (Figure 1A) with a high purity of ≥99% was purchased from LKT Laboratories Inc. (catalog number S8044, St. Paul, MN, USA). Sodium pentobarbital (Sedalphorte^®^) was purchased from Salud y Bienestar Animal S.A. de C.V. (Mexico City, Mexico). Bovine serum albumin (BSA), fatty acid free-BSA (FAF-BSA), 1-chloro-2,4-dinitrobenzene (CDNB), adenosine diphosphate (ADP) sodium salt, adenosine triphosphate (ATP) sodium salt, carbonyl cyanide m-chlorophenylhydrazone (CCCP), cytochrome c (cyt c), safranin O (Saf), sodium succinate dibasic, nitro blue tetrazolium (NBT), decyl-ubiquinone (DUB), reduced decyl-ubiquinone (DUBH_2_), 5,5′-dithiobis-(2-nitrobenzoic acid) (DTNB), 5-thio-2-nitrobenzoic acid (TNB), potassium lactobionate, 2,6-dichlorophenolindophenol sodium salt hydrate (DCPIP), palmitoyl-L-carnitine (PC), L-carnitine, malate (MAL), antimycin A (Ant A), sodium azide, rotenone (Rot), oligomycin (Oligo), sodium L-ascorbate, phenylmethylsulfonyl fluoride (PMSF), Trizma base (Tris), Trizma-hydrochloride (Tris-HCl), Triton X-100, thiamine pyrophosphate, ethylene glycol-bis(2-aminoethyl ether)-N,N,N’,N’-tetra acetic acid (EGTA), Tween-20, 4-(2-hydroxyethyl)-1-piperazine ethane sulfonic acid (HEPES), sodium chloride (NaCl), sodium deoxycholate, sodium dodecyl sulfate (SDS), sodium fluoride (NaF), sodium phosphate dibasic (Na_2_HPO_4_), sodium phosphate monobasic (NaH_2_PO_4_), sodium orthovanadate (Na_3_VO_4_), acrylamide, bis-acrylamide, ammonium persulfate, β-mercaptoethanol, Oil Red O, reduced nicotinamide adenine dinucleotide (NADH) and oxidized NADH (NAD^+^), sodium glutamate, protease, reduced nicotinamide adenine dinucleotide phosphate (NADPH), oxidized NADPH (NADP^+^), glutaraldehyde, sodium cacodylate, osmium tetroxide, uranyl acetate, lead citrate, glycerol, D-mannitol, sucrose, taurine, magnesium chloride (MgCl_2_), protease inhibitor cocktail, bromophenol blue and hematoxylin-eosin (H&E) solution were purchased from Sigma-Aldrich (St. Louis, MO, USA). Ethyl alcohol, potassium hydroxide (KOH), potassium phosphate monobasic (KH_2_PO_4_), and ethylenediaminetetraacetic acid (EDTA) disodium salt dihydrate were purchased from JT Baker (Xalostoc, Ecatepec, Edo. Mexico). Phosphatase inhibitor cocktail was purchased from Roche Applied Science (Mannheim, Germany). Epon resin was purchased from Pelco International (Redding, CA, USA).

### 2.2. Experimental Strategy

Male Wistar rats (*Rattus novergicus*) weighing 200–250 g and aged between 7 and 9 weeks were obtained from Instituto de Fisiología Celular at Universidad Nacional Autónoma de México. The Institutional Animal Care and Use Committee approved the experimental procedures at the Instituto Nacional de Cardiología Ignacio Chávez on September 27 (INC/CICUAL/010/2023). An n = 80 rats were used to carry out experiments and were divided into the following groups: (1) sham (simulated surgery), (2) UUO, conducted, as previously described [12], and maintained for three days, (3) RUUO carried out on day 3, and 4) SFN + RUUO. The “n” used was similar for all groups, and the animals that did not have a correct release of the UUO were replaced. No animals died as a result of the surgery. The success of RUUO was 75% (n = 60), similar to a previous study [18]. A total of 3 rats per group (n = 12) were used for histological evaluation and transmission electron microscopy (TEM); 5–6 rats (n = 24) were used for Western blot assay and triglycerides (TGs) determination. Six rats per group (n = 24) were employed for mitochondrial isolation (employed for ETS, oxygen consumption related to mitochondrial β-oxidation, and ΔΨm). Animal management followed the guidelines for the Care and Use of Laboratory Animals published by Mexican Official Norm Guides (NOM-062-ZOO-1999) [19]. Animals were maintained for a week in plastic rodent caging (Allentown Caging Equipment CO., Inc., Allentown, NJ, USA) at 22 °C with 12 h of light and dark cycles for acclimatization. Food (rodent diet, LAB DIET 5001 from Pet Foods SA DE CV, Mexico City, Mexico) and filtered water (Paraqua-V, Koningin Astridlaan, Belgium) were maintained ad libitum. Afterward, rats were fasted for 12 h before surgery to favor clinical procedures.

For RUUO surgery, the UUO animals were anesthetized with isoflurane (5% induction, 2.0% maintenance), and after disinfecting the ventral area, an incision was performed. The left obstructed ureter was fixed, and in the ureter upper section, a PE-90 coil-shaped catheter was inserted. The distal end of the catheter was inserted into the bladder and secured to the abdominal muscle to prevent dislodgement and allow for proper urine drainage. Urine flow was confirmed through the catheter to ensure the release of the UUO. SFN was given as a preventive treatment (1 mg/kg, i.p.), administered on day 2 after UUO, and continued until day 6 (Figure 1). One SFN dose was selected according to previous findings showing beneficial effects in the UUO [12] and RUUO [10] models. On the seventh day, animals were euthanized by using only a dose of sodium pentobarbital administered intraperitoneally (120 mg/kg, i.p.). In the absence of reflexes, a laparotomy was performed to extract as much blood as possible from the aorta, which guaranteed the death of the animal. The disposal of biological residues was carried out following the NOM-087-SEMARNAT-SSA1-2002 [20].

In total renal cortex tissue, we evaluated histological damage, mitochondrial biogenesis, OXPHOS, FA-binding protein levels, lipid content, and TGs accumulation. In isolated renal mitochondria, we evaluated the activity of mitochondrial complexes of the ETS, bioenergetics, ΔΨm related to β-oxidation, mitochondrial dynamics, ultrastructure damage, mitophagy, and autophagic bodies (Figure 1). All experiments were carried out in a randomized and blinded manner.

### 2.3. Renal Histology and Mitochondrial Ultrastructure by TEM

Kidneys were longitudinally sectioned, and one half was fixed by immersion in 10% formaldehyde dissolved in phosphate buffer saline (PBS) pH 7 for 24 h. Sections of 3 μm thickness were stained with H&E to assess kidney damage and with Masson’s trichrome solution to evaluate fibrosis. Fibrosis was quantified using the ImageJ software version 1.54k (U.S. National Institutes of Health, Bethesda, MD, USA). The remaining kidney halves were immediately frozen in liquid nitrogen, and cryostat sections were prepared and stained with Oil Red O to visualize lipid accumulation.

For TEM, a 1 µm section of the renal cortex was initially fixed by immersion in a fixing solution. The tissue was then fragmented, fixed in 4% glutaraldehyde in 0.1 M cacodylate buffer (pH 7), and postfixed in 2% osmium tetroxide in 100 mM cacodylate buffer. Following dehydration with increasing ethanol concentrations, the fragments were gradually infiltrated with Epon resin. Ultrathin sections (60–80 nm) were cut, counterstained with saturated uranyl acetate and Reynolds leads citrate solution, and examined using an FEI Technai transmission electron microscope (Thermo Fisher Scientific, Waltham, MA, USA).

### 2.4. Protein Extraction, Quantification, and Western Blot Assays

As previously reported, protein extraction and quantification were performed [12]. Briefly, 100 mg of kidney tissue was homogenized with a polytron homogenizer (Kinematica AG, Malters, Switzerland) in 1 mL of radioimmunoprecipitation assay (RIPA) buffer (40 mM Tris-HCl, 150 mM NaCl, 2 mM EDTA, 1 mM EGTA, 5 mM NaF, 1 mM Na_3_VO_4_, 1 mM PMSF, 0.5% sodium deoxycholate, 0.1% SDS, pH 7.6) and supplemented with protease and phosphatase inhibitor cocktails. The samples were centrifuged at 15,000× *g* for 20 min, and the proteins in the supernatant were quantified using the Lowry method [21]. Samples were prepared at a 1:1 ratio with 2× Laemmli buffer (65.8 mM Tris-HCl, pH 6.8, 2% SDS, 0.7 mM β-mercaptoethanol, 20% glycerol, and 0.01% bromophenol blue) and heated for 10 min at 95 °C. Then, samples were used for sodium dodecyl sulfate-polyacrylamide gel electrophoresis (SDS-PAGE) and Western blot assays, as previously reported [10]. Proteins analyzed by Western blot were associated with fibrosis [fibronectin (FN), collagen IV (Col IV), alpha-smooth muscle protein (α-SMA)], biogenesis [peroxisome proliferator-activated receptor γ co-activator-1α (PGC-1α), the nuclear respiratory factor (NRF1)], mitochondrial mass [voltage-dependent anion channel (VDAC), mitochondrial transcription factor A (TFAM), carnitine palmitoyl acyl transferase 1A (CPT1A)], protein levels of subunits of OXPHOS [NADH: ubiquinone oxidoreductase subunit B8 (NDUFB8), succinate dehydrogenase complex iron-sulfur subunit B (SDHB), ubiquinol-cytochrome c reductase core protein 2 (UQCRC2), mitochondrially encoded cytochrome c oxidase 1 (MTCO1), the ATP synthase-α subunit (ATP5A)], synthesis of TGs [diacylglycerol acyl transferase 1 (DGAT1)], uptake of TGs [cluster of differentiation 36 (CD36), and storage of TGs (peroxisome proliferator-activated receptor-γ (PPAR-γ)]. Proteins related to mitochondrial dynamics [dynamin-related protein 1 (DRP1), mitofusin 1 and 2 (MFN1 and MFN2)], mitophagic flux [phosphatase, tensin homolog deleted on chromosome 10 (PTEN)-induced kinase 1 (PINK1) and parkin], autophagic flux [beclin, sequestosome 1 (p62), as well as the microtubule-associated proteins 1A/1B light chain 3 (LC3-II/LC3-I), were also evaluated.

The sources of antibodies employed for Western blot assays are found in Supplementary Table S1.

### 2.5. Renal Mitochondria Isolation

Immediately after killing rats, one-third of the kidney cortex was immersed in a cooled PBS pH 7.4. Then, the kidney was cut into small pieces and homogenized in isolation buffer containing 225 mM D-mannitol, 75 mM sucrose, 1 mM EDTA, 5 mM HEPES, 0.1% BSA, pH 7.4 at 4 °C with a Glass/Teflon Potter Elvehjem tissue grinder (Sigma-Aldrich, St. Louis, MO, USA). The resulting supernatant was then subjected to sequential differential centrifugation, as described previously [12], and the pellet was resuspended in 180 μL of FAF-BSA isolation buffer.

### 2.6. Activity of the Four Mitochondrial Complexes of the ETS: Complex I (CI), Complex II (CII), Complex III (CIII), and Complex IV (CIV)

The activity of the mitochondrial complexes of the ETS was evaluated using 20 μg of protein from freshly isolated mitochondria, as previously reported [12], and utilizing a Synergy HT microplate reader (Biotek Instruments Inc., Winooski, VT, USA) at 37 °C. Briefly, CI activity was determined at 600 nm by following DUB reduction to reduced decyl ubiquinone (DUBH_2_) in a reaction linked to the disappearance of DCPIP, proportional to CI activity, using NADH as a substrate. The reaction was carried out in the presence of 2.5 μM Ant A and 1 mM sodium azide. The non-specific CI activity was detected by adding 2.5 μM Rot. CII activity was evaluated following the disappearance of DCPIP in a reaction mix containing 10 mM succinate, 2.5 μM Rot, and 2.5 μM Ant A. Then, 5 mM MAL was added to assess the non-specific CII activity. CIII activity was determined at 550 nm by following the reduction in cyt c, employing DUBH_2_ as a substrate. The non-specific activity was measured by adding 2.5 μM Ant A. The activity of CIV was determined by following the oxidation of cyt c at 565 nm, and the non-specific activity was evaluated using 1 mM sodium azide. The activity of the mitochondrial complexes was expressed as nmol per minute per milligram of protein (nmol/min/mg protein).

### 2.7. Oxygen Consumption Related to Mitochondrial β-Oxidation

As previously reported, oxygen consumption was evaluated in freshly isolated mitochondrial fractions [22]. Briefly, 2 mL of mitochondrial respiration buffer MiR05 (0.5 mM EGTA, 20 mM taurine, 3 mM MgCl_2_, 60 mM potassium lactobionate, 10 mM KH_2_PO_4_, 20 mM HEPES, 110 mM sucrose, and 1 g/L FAF-BSA, pH 7.4) were loaded into 2 mL chamber of high-resolution respirometer (Oxygraph O2k, Oroboros Instruments GmbH, Innsbruck, Austria) at 37 °C. Electron transport began with oxidation-linked substrates (2 mM L-carnitine, 2 μM PC plus 2 mM MAL), known as state (S) 2 (S2). S3 was stimulated by adding 2.5 mM ADP, while S4 was induced by adding 2.5 μM oligo (S4o). The parameters were corrected by adding 0.5 μM Rot plus 2.5 μM Ant A to obtain residual respiration. Respiratory control (RC) was obtained using the S3/S4o ratio, and OXPHOS-associated respiration (P) was obtained by subtracting S3-S4o.

### 2.8. ΔΨm Related to β-Oxidation

The changes in ΔΨm were evaluated in isolated fresh mitochondria using an O2k-Fluorometer (Oroboros Instruments GmbH) at 37 °C, as previously reported [18]. Briefly, 5 μM Saf was used as a probe to measure ΔΨm. The respective substrates, 2 mM L-carnitine, 2 μM PC plus 2 mM MAL, were added to stimulate β-oxidation (S2). S3 was obtained by adding 2.5 mM ADP, and S4o was determined by adding 2.5 μM oligo. Thus, ΔΨm was calculated in the different respiratory states (S2, S3, and S4o). A total of 500 nM Rot plus 2.5 μM Ant A were added to dissipate ΔΨm and correct the non-specific interactions. Data are represented as a change in Saf concentration and normalized per milligram of protein.

### 2.9. TGs Determination

TGs were determined using a commercial kit (Triglyceride-SL, Sekisui Diagnostics LLC, Burlington, MA, USA) following the Folch extraction method with modifications [23]. The results were expressed as mg TGs/mg of protein.

### 2.10. Statistical Analysis

The results were expressed as mean ± standard error of the mean (SEM). Data were analyzed using GraphPad Prism 8 software (GraphPad Software, Boston, MA, USA, www.graphpad.com, accessed on 27 November 2023). Normality Shapiro–Wilk tests were applied to determine whether data followed a normal distribution. Then, the analysis of variance (ANOVA) was used for multiple comparisons, followed by Tukey’s test. Significant differences between groups were set at *p* < 0.05.

## 3. Results

### 3.1. SFN Mitigates Renal Structural Damage and Fibrosis After RUUO

The present study confirmed that SFN pre-administration reduced the renal damage observed in the RUUO model [10]. The kidneys of the UUO group exhibited extensive tubular damage with numerous convoluted proximal tubules related to atrophy or cell death (Figure S1A,B). In contrast, the RUUO group improved renal tubular damage (Figure S1C), an effect further enhanced in the SFN + RUUO group (Figure S1D).

**Figure 2:**
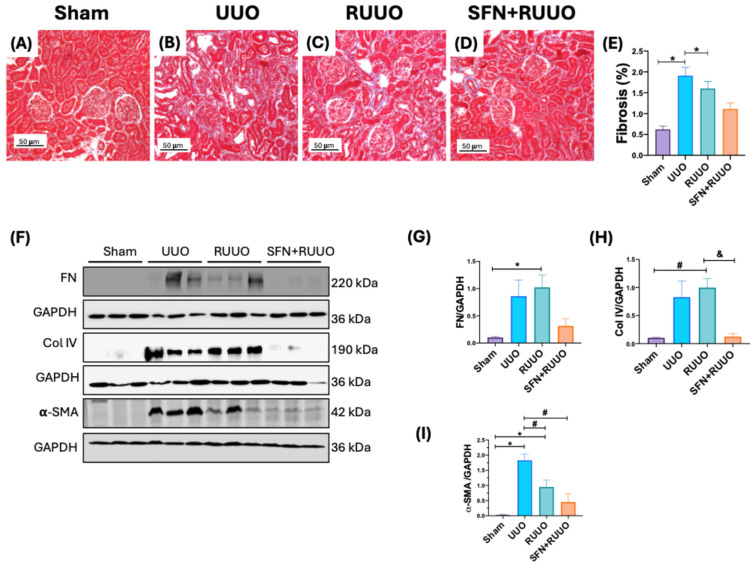
Sulforaphane (SFN) pre-administration effects on renal fibrosis in the release of unilateral ureteral obstruction (RUUO) model. Representative histological micrographs of kidneys stained with Masson’s trichrome staining. (**A**) The Sham group did not show fibrosis. (**B**) In the unilateral ureter obstruction (UUO) group, notable interstitial fibrosis was shown in blue. (**C**) There was a notable decrease in interstitial fibrosis in the RUUO group. (**D**) The decrease in fibrosis is even more evident in the SFN + RUUO group. (**E**) Quantitative analysis of fibrosis. n = 3, * *p* < 0.05 vs. sham group. Scale bar: 50 μm. (**F**) Representative immunoblots and densitometric analysis for (**G**) fibronectin (FN), (**H**) collagen IV (Col IV), and (**I**) alpha-smooth muscle actin (α-SMA). Glyceraldehyde 3-phosphate dehydrogenase (GAPDH) was used as loading control. All data were analyzed by one-way ANOVA followed by Tukey’s test and expressed as mean ± SEM, n = 6 per group. * *p* < 0.05 vs. sham, ^#^ *p* < 0.05 vs. UUO, ^&^ *p* < 0.05 vs. RUUO. Sham: simulated surgery; UUO: unilateral ureteral obstruction; RUUO: release of UUO; SFN + RUUO: SFN administered before RUUO. kDa = kilodaltons.

In addition to renal damage for H&E, we evaluated fibrosis, a characteristic of the UUO model that has been reported to continue and even increase after RUUO [6,24]. Therefore, we performed the Masson’s trichrome stain to evaluate the occurrence of fibrosis. Micrographs revealed focal areas of interstitial fibrosis (blue staining) in the UUO group compared to sham (Figure 2A,B). In contrast, the RUUO group showed a decrease in these fibrotic areas (Figure 2C,E), but notably, the treatment with SFN further reduced interstitial fibrosis, surpassing the improvement seen with the RUUO procedure alone (Figure 2D,E). These findings suggested that SFN partially mitigated kidney damage and fibrosis in the RUUO model.

To further confirm the fibrosis reduction conferred by SFN treatment, we evaluated the renal protein levels of FN, Col IV, and α-SMA by immunoblot assays. FN and Col IV levels tended to increase in the UUO group, compared with the sham group (Figure 2F–H), although this change was not significant. In contrast, α-SMA levels suffered a considerable increase in the UUO group compared to sham animals (Figure 2F,I). Regarding Col IV and FN, both proteins continued to be significantly elevated in the RUUO group, and only SFN treatment could reduce them. However, Col IV’s effect was only statistically significant (Figure 2F–H). Regarding α-SMA, the RUUO group showed a significant decrease in its levels compared to UUO, which were even lower than in the SFN + RUUO group (Figure 2F,I). These findings confirm the superior anti-fibrotic effect of SFN treatment in the RUUO model.

### 3.2. SFN Enhances Mitochondrial Biogenesis and Mass in the RUUO Model

**Figure 3:**
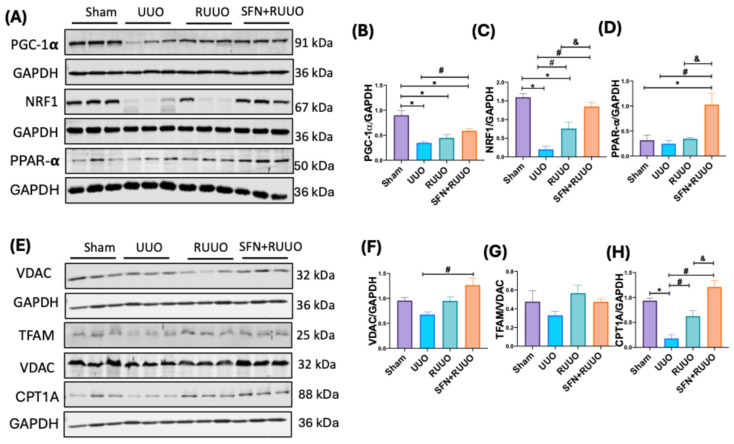
Sulforaphane (SFN) induces mitochondrial biogenesis and increases mitochondrial mass after the release of the unilateral ureteral obstruction (RUUO) model. (**A**) Representative immunoblots and densitometric analysis for (**B**) peroxisome proliferator-activated receptor γ co-activator-1α (PGC-1α), (**C**) nuclear respiratory factor 1 (NRF1), and (**D**) peroxisome proliferator-activated receptor-α (PPAR-α). (**E**) Representative immunoblots and densitometric analysis for (**F**) voltage-dependent anion channel (VDAC), (**G**) mitochondrial transcription factor A (TFAM), and (**H**) carnitine palmitoyl acyl transferase 1A (CPT1A). Glyceraldehyde 3-phosphate dehydrogenase (GAPDH) was employed as loading control. VDAC was used as loading control for TFAM. Data were analyzed by one-way ANOVA followed by Tukey’s test and expressed as mean ± SEM. n = 6 per group. * *p* < 0.05 vs. sham, ^#^ *p* < 0.05 vs. UUO, ^&^ *p* < 0.05 vs. RUUO. Sham: simulated surgery; UUO: unilateral ureteral obstruction; RUUO: release of UUO; SFN + RUUO: SFN administered before RUUO.

Previous studies have suggested that fibrosis development in UUO conditions might be attributed to a decrease in mitochondrial biogenesis [12,14,25]. While RUUO improves tubular health, the impact on biogenesis remains unclear. To investigate the effect of RUUO on mitochondrial biogenesis and to assess the potential role of SFN, we measured the protein levels of both PGC-1α and NRF1, the key regulators of mitochondrial biogenesis [26]. Similar to previous reports [12,14], PGC-1α and NRF1 levels decreased in the UUO group compared to the sham (Figure 3A–C). Notably, the RUUO group had no significant effect on PGC-1α levels, but SFN administration did (Figure 3A,B). Regarding NRF1, the RUUO led to a partial recovery of this protein, notably enhanced by SFN treatment until similar levels to the sham group (Figure 3A,C). These data suggest an effect on mitochondrial biogenesis mediated by SFN treatment.

We also evaluated the levels of peroxisome proliferator-activated receptor-α (PPAR-α), which is involved in FA metabolism to regulate energy homeostasis [26,27] and also interacts with PGC-1α. Although no differences were observed between sham, UUO, and RUUO groups (Figure 3A,D), we observed a significant increase in PPAR-α levels in the SFN + RUUO group, suggesting that SFN regulated PGC-1α coactivators during RUUO.

To determine if the enhancement of mitochondrial biogenesis could increase mitochondrial mass, we evaluated the protein levels of VDAC, an outer mitochondrial membrane (OMM) protein [28], and TFAM, a protein involved in mitochondrial deoxyribonucleic acid (mtDNA) maintenance [29]. While VDAC levels showed a non-significant decrease in the UUO group, the release of ureteral obstruction (RUUO group) tended to recover VDAC levels, and SFN (SFN + RUUO group) caused an even greater increase, which was significant (Figure 3E,F). Like VDAC, the evaluation of TFAM levels also showed a non-significant decrease in the UUO group and a recovery in the RUUO group. However, no effect was observed with the use of SFN. Still, no significant differences were observed between the groups (Figure 3E–G). These data show that SFN improves the effect of the release of UUO regarding mitochondrial mass.

In addition, we also determined the protein levels of CPT1A, the limiting enzyme in mitochondrial β-oxidation of FA, regulated by PPAR-α [30]. We found a significant decrease in CPT1A levels in animals with UUO compared to sham rats (Figure 3E,H). Moreover, CPT1A levels were significantly reduced in the RUUO group, indicating an improvement after obstruction removal. Furthermore, SFN significantly increased CPT1A levels further than in the RUUO group (Figure 3E,H). These findings suggest that SFN exerts a more pronounced effect on CPT1A levels, potentially mediated through increased PPAR-α expression. In addition, SFN enhances mitochondrial biogenesis and upregulates key coactivators, increasing mitochondrial mass and the expression of proteins involved in β-oxidation of FA in the RUUO model.

### 3.3. SFN Enhances CII and CIII Activities in Isolated Mitochondria

**Figure 4:**
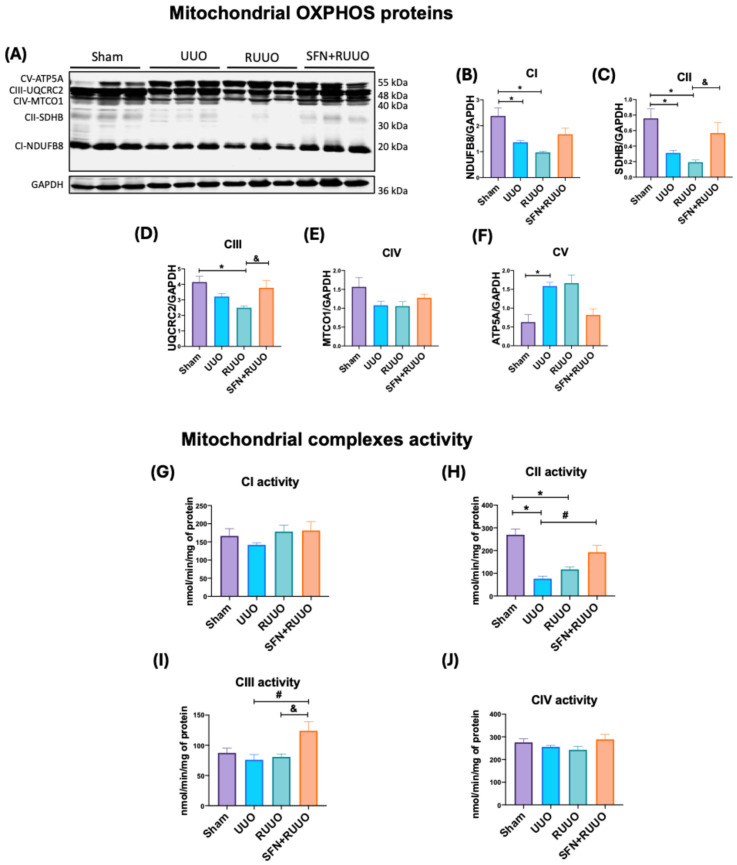
Sulforaphane (SFN) affects mitochondrial complexes (CI, CII, CIII, CIV, and CV). (**A**) Representative immunoblot and densitometric analysis of (**B**) NADH: ubiquinone oxidoreductase subunit B8 (NDUFB8), (**C**) succinate dehydrogenase complex iron-sulfur subunit B (SDHB), (**D**) ubiquinol–cytochrome c reductase core protein 2 (UQCRC2), (**E**) mitochondrially encoded cytochrome c oxidase 1 (MTCO1), (**F**) Adenosine triphosphate (ATP) synthase-α subunit (ATP5A). Glyceraldehyde 3-phosphate dehydrogenase (GAPDH) was employed as a loading control for immunoblots. n = 6 per group. Activities of (**G**) complex I (CI), (**H**) complex II (CII), (**I**) complex III (CIII), and (**J**) complex IV (CIV) were evaluated in isolated mitochondria for all groups. n = 4–5 per group. Data were analyzed by one-way ANOVA followed by Tukey’s test. They were expressed as mean ± SEM, * *p* < 0.05 vs. sham, ^#^ *p* < 0.05 vs. UUO, ^&^ *p* < 0.05 vs. RUUO. Sham: simulated surgery; UUO: unilateral ureteral obstruction; RUUO: release of UUO; SFN + RUUO: SFN administered before RUUO.

To further corroborate the increase in mitochondrial mass, we also assessed the relative protein levels of subunits within the five mitochondrial complexes of the ETS [31]. In the UUO group, we found a significant reduction in the NDUFB8 and SDHB protein levels of the CI and CII, compared with sham (Figure 4A–C). Additionally, the levels of UQCRC2 and MTCO1, proteins of the CIII and CIV, respectively, tended to reduce non-significantly (Figure 4A,D,E). While the reductions in CI, CII, CIII, and CIV levels persisted in the RUUO group, SFN treatment significantly prevented the changes in CII and CIII (Figure 4A,C,D). A non-significant increase in the levels of CI and CIV was observed (Figure 4A,B,E). Regarding ATP5A, we observed elevated levels in both UUO and RUUO groups (Figure 4A,F). Although SFN treatment demonstrated a trend toward restoring ATP5A levels to those observed in the sham group, it was not statistically significant (Figure 4A,F). These findings suggest that SFN contributes to restoring some mitochondrial complex subunits in the RUUO model.

To investigate if the SFN-induced restoration of protein levels of mitochondrial complexes of the ETS might be reflected in their activities, we evaluated this parameter in CI, CII, CIII, and CIV in isolated mitochondria. The activities of CI and CIV were unchanged in all experimental groups (Figure 4G,J). Conversely, CII activity was significantly reduced in the UUO and RUUO groups compared to the sham group (Figure 4H). SFN treatment, however, mitigated this reduction, similar to its effect on SDHB protein levels, suggesting an overall improvement in CII function. Although CIII activity was neither affected in UUO nor RUUO groups compared to the sham group, a notable enhancement in the activity of this complex was induced by SFN (Figure 4I). Therefore, our results suggest that SFN promotes the enhancement of CII and CIII activities in the RUUO model.

### 3.4. SFN Restores the Oxygen Consumption Associated with β-Oxidation in the RUUO Model

**Figure 5:**
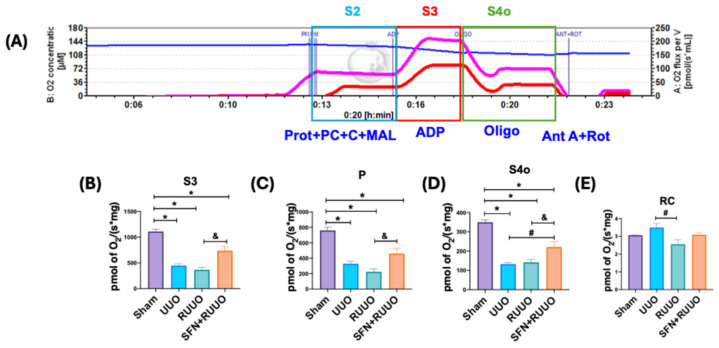
Sulforaphane (SFN) partially restores mitochondrial oxygen consumption associated with β-oxidation during the release of the unilateral ureteral obstruction (RUUO) model. (**A**) Representative trace of an experiment tracking the oxygen (O_2_) consumption rate by adding different substrates to enhance the different respiratory states. The blue line shows oxygen concentration; the pink line shows the oxygen consumption rate of isolated renal mitochondria of a rat from the sham group, and the red line shows the oxygen consumption rate of isolated renal mitochondria of a rat from the RUUO group. Oxygen consumption begins by adding proteins from isolated renal mitochondria and the substrates palmitoyl-L-carnitine, carnitine, and malate (Prot + PC + C + MAL) [State 2 (S2)]. (**B**) State 3 (S3) was obtained by adding adenosine diphosphate (ADP); (**C**) oxidative phosphorylation associated respiration (P) was obtained by subtracting S3 minus S4o; (**D**) State 4 (S4o) was obtained by adding oligomycin (Oligo). (**E**) Respiratory control (RC) was reached with rotenone (Rot) and antimycin A (Ant A) additions. Data were analyzed by one-way ANOVA followed by Tukey’s test, and they were expressed as mean ± SEM, n = 6 per group. * *p* < 0.05 vs. sham, ^#^ *p* < 0.05 vs. UUO, ^&^ *p* < 0.05 vs. RUUO. Sham: simulated surgery; UUO: unilateral ureteral obstruction; RUUO: release of UUO; SFN + RUUO: SFN administered before RUUO.

To elucidate whether the reduction in mitochondrial biogenesis and mitochondrial complexes activities of the ETS observed in the UUO model impacted renal mitochondrial ATP production and to assess the potential ameliorative effects of SFN on this process, we measured the β-oxidation-related respiratory parameters in isolated renal mitochondria (Figure 5A). Respiratory parameters S3 (respiration coupled with ATP production), P, and S4o decreased in all groups of UUO compared to sham animals (Figure 5B,D). SFN administration significantly increased S3 and P compared to the RUUO group and S4o compared to the UUO group (Figure 5B–D), indicating upregulation of β-oxidation-related ATP production. RC tended to increase in the UUO group compared to sham animals, but it was significantly decreased in the RUUO group compared to the UUO group (Figure 5E). The SFN + RUUO group did not differ significantly from any other group, suggesting that RC was unaffected by SFN administration (Figure 5E). Collectively, these results indicate that SFN partially restores β-oxidation in the RUUO model.

### 3.5. SFN Does Not Affect ΔΨm Associated with β-Oxidation

**Figure 6:**
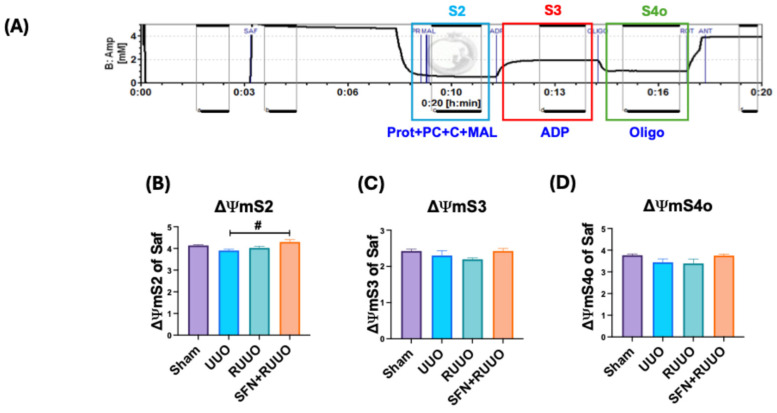
Sulforaphane (SFN) did not affect the mitochondrial membrane potential (ΔΨm) related to β-oxidation. (**A**) Representative trace of an experiment tracking the ΔΨm in the different states (S) by adding different substrates using safranin (Saf) as a probe. (**B**) ΔΨm for state 2 (ΔΨmS2) was obtained by adding protein mitochondria, palmitoyl-L-carnitine, carnitine, and malate (Prot + PC + C + MAL). (**C**) ΔΨm for state 3 (ΔΨmS3) was obtained by adding adenosine diphosphate (ADP), and (**D**) ΔΨm for state 4o (ΔΨmS4o) was obtained by adding oligomycin (Oligo). Data were analyzed by one-way ANOVA followed by Tukey’s test and expressed as mean ± SEM, n = 6 per group. ^#^ *p* < 0.05 vs. UUO. Sham: simulated surgery; UUO: unilateral ureteral obstruction; RUUO: release of UUO; SFN + RUUO: SFN administered before RUUO.

We evaluated the changes in ΔΨm in isolated renal mitochondria using Saf as a probe to determine if ΔΨm was altered due to mitochondrial disturbances and the possible SFN effect (Figure 6A). We did not find differences between the sham, UUO, and RUUO groups for ΔΨmS2. Only a slight increase in this state was observed for the SFN + RUUO group (Figure 6B). For ΔΨmS3 and ΔΨmS4o states, we did not find statistical differences among the groups (Figure 6C,D). These data suggested that ΔΨm was neither altered during ureteral obstruction (UUO group) nor after ureteral obstruction release (RUUO group), indicating the maintenance of mitochondrial membrane potential.

### 3.6. SFN Decreases FA Uptake, Intrarenal TGs, and Lipid Deposition in the RUUO Model

**Figure 7:**
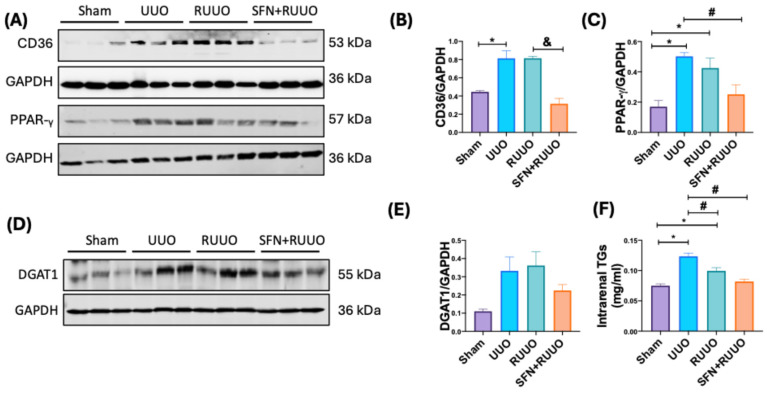
Sulforaphane (SFN) regulates fatty acid (FA) uptake and decreases lipids and triglycerides (TGs) in the release of unilateral ureteral obstruction (RUUO) model. (**A**) Representative immunoblots and densitometric analysis of (**B**) cluster of differentiation 36 (CD36), (**C**) peroxisome proliferator-activated receptor-γ (PPAR-γ). (**D**) Representative immunoblots and densitometric analysis of (**E**) diacylglycerol acyl transferase 1 (DGAT1). Glyceraldehyde 3-phosphate dehydrogenase (GAPDH) was a loading control, n = 6. (**F**) Intrarenal TGs were evaluated in the renal cortex for all experimental groups. Data are expressed as mean ± SEM and were analyzed by one-way ANOVA followed by Tukey’s test. * *p* < 0.05 vs. sham, ^#^ *p* < 0.05 vs. UUO, ^&^ *p* < 0.05 vs. RUUO. Sham: simulated surgery; UUO: unilateral ureteral obstruction; RUUO: release of UUO; SFN + RUUO: SFN administered before RUUO.

The energetic disbalance caused by mitochondrial β-oxidation reduction promotes the upregulation of proteins involved in FA uptake, synthesis, and storage [32,33,34]. To elucidate the possible alterations in this process, we determined the levels of proteins involved in regulating FA metabolism and its use. Then, we evaluated the protein levels of CD36, the primary receptor involved in FA uptake found in the cell membrane. Similar to previous data, CD36 was upregulated in UUO compared to the sham group [35,36]. The upregulation of this receptor continued to increase after ureteral obstruction release and decreased in the SFN + RUUO group compared to the UUO and RUUO groups (Figure 7A,B), suggesting an attenuation of FA uptake. Likewise, the PPAR-γ levels, a protein involved in FA storage and synthesis, increased in the UUO group and remained high in the RUUO group (Figure 7A,C). Conversely, PPAR-γ levels decreased in the SFN-treated group comparable to sham animals and were significantly lower than in the UUO and RUUO groups (Figure 7A,C).

We assessed renal lipid accumulation through Oil Red-O staining to investigate the SFN effect in the dysregulation of lipid metabolism. Numerous lipid-laden cytoplasmic vacuoles were detected in the UUO group compared to the sham group (Figure S2A,B). Notably, the RUUO group exhibited fewer of these vacuoles (Figure S2C), and this reduction was even more pronounced in the SFN + RUUO group (Figure S2D), suggesting that SFN avoided lipid accumulation. To support the latter, we evaluated the synthesis and amount of renal TGs (Figure 7D–F). Thus, we determined the protein levels of DGAT1, a key enzyme involved in TGs biosynthesis. We found a non-significant increase in this protein in the UUO group, which was not reduced by the RUUO procedure (Figure 7D,E). Although not statistically significant, we observed that DGAT1 decreased in the SFN + RUUO group (Figure 7D,E), suggesting an attenuation in TGs synthesis. To corroborate this, the assessment of TGs content in renal tissues revealed a significant increase in the obstructed kidney, which was slightly mitigated in the RUUO group. Notably, TGs accumulation was prevented by SFN pretreatment, surpassing the effect of RUUO alone (Figure 7D,F). These findings indicate that SFN mitigates renal lipid deposition, such as TGs in the RUUO model.

### 3.7. SFN Restores Mitochondrial Dynamics in the RUUO Model

**Figure 8:**
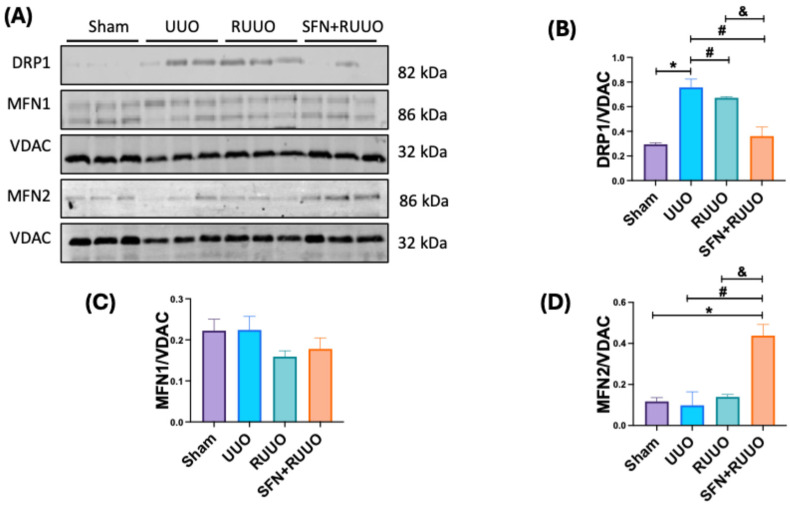
Sulforaphane (SFN) mitigates mitochondrial fission and increases mitochondrial fusion after the release of the unilateral ureteral obstruction (RUUO). (**A**) Representative immunoblots and densitometric analysis of (**B**) dynamin-related protein 1 (DRP1), (**C**) Mitofusin 1 (MFN1), and (**D**) Mitofusin 2 (MFN2). Voltage anion-dependent channel (VDAC) was used as the loading control. Data were analyzed by one-way ANOVA, followed by Tukey’s test, and they are expressed as mean ± SEM, n = 3 per group. * *p* < 0.05 vs. sham, ^#^ *p* < 0.05 vs. UUO, ^&^ *p* < 0.05 vs. RUUO. Sham: simulated surgery; UUO: unilateral ureteral obstruction; RUUO: release of UUO; SFN + RUUO: SFN administered before RUUO.

Dysregulation of OXPHOS and lipid accumulation disrupt mitochondrial dynamics, often leading to increased mitochondrial fission and decreased fusion [37]. To assess the modifications of these processes we evaluated the levels of mitochondrial fission protein DRP1 as well as mitochondrial fission proteins MFN1 and MFN2 [38]. We observed that DRP1 levels were higher in UUO, which were reduced after obstruction release (RUUO group). SFN pretreatment attenuated this increase even more, suggesting the reduction in mitochondrial fission (Figure 8A,B). While MFN1 and MFN2 levels were unaffected by UUO or RUUO (Figure 8A,C,D), SFN treatment only significantly increased MFN2 expression (Figure 8A,D). These findings indicate that SFN restores the balance in mitochondrial dynamics in the RUUO model by lessening mitochondrial fission and partially promoting the mitochondrial fusion process.

### 3.8. SFN Mitigates Mitochondrial Structural Damage and Restores Mitophagy and Autophagy Flux in the RUUO Model

**Figure 9:**
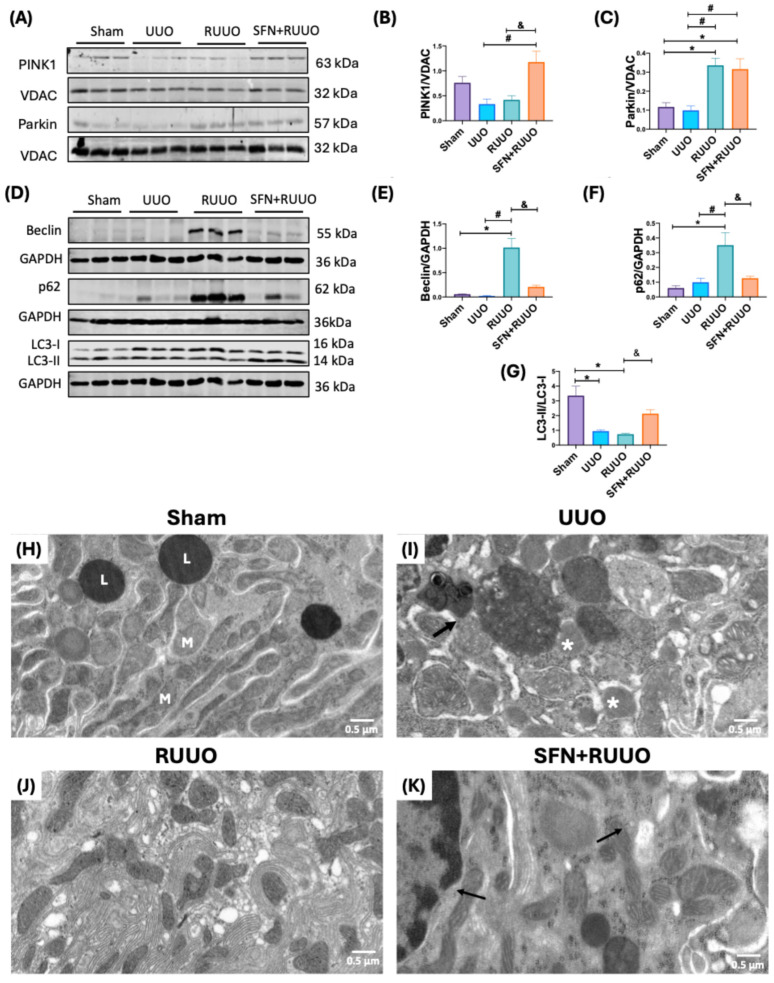
Prevention of mitophagy and autophagy alterations mediated by sulforaphane (SFN) in the unilateral ureteral obstruction (RUUO) model. (**A**) Representative immunoblots of mitophagy proteins. Densitometric analyses of (**B**) phosphatase and tensin homolog deleted on chromosome 10 (PTEN)-induced kinase 1 (PINK1) and (**C**) Parkin. Voltage anion-dependent channel (VDAC) was used as the loading control. Data are expressed as mean ± SEM and were analyzed by one-way ANOVA followed by Tukey’s test. n = 6. (**D**) Representative immunoblots of autophagy proteins. Densitometric analysis of (**E**) Beclin, (**F**) Sequestosome 1 (p62), and (**G**) microtubule-associated proteins 1A/1B light chain 3 (LC3-II/LC3-I) ratio. Glyceraldehyde 3-phosphate dehydrogenase (GAPDH) was employed as a loading control. Regarding the ultrastructure of convoluted proximal cells, the electron microscopy micrographs show normal morphology with numerous elongated or spherical mitochondria (M) and electron-dense round vacuoles corresponding to lysosomes (L) of a sham rat (image (**H**)). Large lysosomes, some of them fused with mitochondria corresponding to mitophagy (arrow), and many mitochondria with irregular shape and cristae effacement (white asterisks) are observed in a rat with UUO (image (**I**)). Numerous irregularly shaped mitochondria with preserved cristae surrounded by small empty vacuoles and extensively developed rough endoplasmic reticulum are seen in tubular epithelial cells from a rat with RUUO (image (**J**)). Elongated fused mitochondria (arrows) with well-preserved cristae surrounded by numerous free ribosomes are observed in tubular epithelial cells from a rat treated with SFN + RUUO (image (**K**)). Data were analyzed by one-way ANOVA followed by Tukey’s test, and they were expressed as mean ± SEM, n = 6 per group. * *p* < 0.05 vs. sham, ^#^ *p* < 0.05 vs. UUO, ^&^ *p* < 0.05 vs. RUUO. Sham: simulated surgery; UUO: unilateral ureteral obstruction; RUUO: release of UUO; SFN + RUUO: SFN administered before RUUO.

Excessive mitochondrial fission commonly leads to mitophagy activation [39]. This mechanism involves the recruitment of PINK1 at the OMM through DRP1, which targets Parkin and initiates mitophagy [40]. Although not statistically significant, PINK1 levels were decreased in the UUO group and remained low in the RUUO group. However, SFN treatment significantly increased PINK1 expression (Figure 9A,B). Parkin levels remained unchanged in the UUO group compared to sham animals but were significantly increased in the RUUO and SFN + RUUO groups (Figure 9A,C).

To investigate whether altered mitophagy could activate autophagy, another process involved in selectively degrading damaged mitochondria [41] and frequently disrupted in the UUO model [12,16,42], we evaluated the levels of autophagy-related proteins after ureteral obstruction release, as well as the effect of SFN treatment in this mechanism. The levels of Beclin and p62 were not affected in the UUO group compared to the sham group (Figure 9D–F). Notably, the RUUO group exhibited elevated levels of both proteins, suggesting the induction and the posterior impairment of autophagy flux (Figure 9D–F). Additionally, the microtubule-associated proteins 1A/1B light chain 3 (LC3-II/LC3-I) ratio was significantly reduced in the UUO and RUUO groups, supporting the idea that autophagy flux was stopped (Figure 9D,G). The recovery of p62 levels and LC3-II/LC3-I ratio by SFN treatment (Figure 9F,G) suggests that obstruction release impairs autophagic flux, a defect rescued by SFN treatment. 

In addition to biochemical assessments of mitochondrial function, we examined mitochondrial morphology and ultrastructural abnormalities in the mitochondria-rich epithelial cells of the proximal convoluted tubules. In the sham group, TEM showed well-preserved mitochondria with electron-dense vacuoles corresponding to lysosomes (Figure 9H). Proximal tubules in the UUO group exhibited a reduced number of mitochondria, characterized by irregular shapes, frequent cristae disruption, enlarged lysosomes, and the presence of mitophagosomes formed by the fusion of lysosomes with damaged mitochondria (Figure 9I). RUUO group exhibited lesser abnormalities, such as mitochondria with irregular shape and size but with well-preserved cristae, surrounded by small empty vacuoles and well-developed endoplasmic reticulum (Figure 9J). In contrast, the SFN + RUUO group displayed significantly fewer abnormalities, with abundant fused mitochondria exhibiting well-preserved cristae and a reduced number of intact lysosomes (Figure 9K). These findings further support that SFN treatment improves mitochondrial morphology and restores autophagy in the RUUO model.

## 4. Discussion

It has been reported that RUUO partially restores kidney function after UUO [9], and pre- or concurrent treatments during the ureteral obstruction release process can give greater efficacy in mitigating the pathological and molecular pathways induced by UUO [24,43,44]. Therapeutic approaches have traditionally targeted arterial pressure and body fluids volume regulating the renin-angiotensin system (RAS) and the inflammatory pathways [24,45,46] with a limited focus on antioxidants. Notably, our previous work in the RUUO model demonstrated that the antioxidant SFN at 1 mg/kg activates Nrf2, promoting antioxidant response and reducing renal damage after UUO release [10]. The renal function recovery was observed through the restoration of the creatinine renal function marker and the reduction in kidney injury molecule-1 (KIM-1) levels, indicating that SFN improves kidney functionality [10]. These observations coincide with other studies showing that SFN provides antioxidant benefits in distinct pathologies using the same dose [47,48,49,50]. However, one limitation of our study might be related to the use of only one dose of SFN because the use of more doses will allow us to investigate if SFN effects are dose-dependent, increased, or canceled. The latter could be of clinical relevance because, to our knowledge, no antioxidant therapy has been utilized as a pretreatment before UUO release surgery. In addition, SFN-induced Nrf2 activation also restored the mitochondrial S-glutathionylation process in the RUUO model [10], suggesting a protective mechanism in mitochondria. Thus, in the present study, we focused on determining mitochondrial parameters related to mitochondrial biogenesis, bioenergetics, dynamics, and mitophagy/autophagy under the same conditions as an established model.

SFN was administered the second day after UUO surgery based on previous observations. For instance, the mitochondrial protective effect of SFN in UUO was found when administrating this antioxidant after UUO (day two), which found an improvement in mitochondrial biogenesis and decreased fibrosis in UUO [12]. In addition, in the RUUO model, the decrease in oxidative stress and inflammation was found under similar conditions of SFN administration, ultimately avoiding apoptosis [10]. Other treatments in the RUUO model also employ similar administration schemes before removing UUO. For instance, allopurinol was given to the rats twenty minutes before obstruction relief [51]. On the other hand, diclofenac sodium was administered immediately after obstruction surgery and maintained up to sixteen days after reversal obstruction [43]. Both studies showed an amelioration in renal damage and function, suggesting that treatments were effective when they were administered after UUO surgery. On the other hand, SFN might be given after removing UUO and have beneficial effects. The latter mimics what happens in clinics, mainly in pediatric patients suffering prenatal ureteral obstruction, where treatments are given after surgery release [52]. Therefore, including studies involving SFN treatment after RUUO focused on pediatric management might be relevant.

In our model, we effectively observed the potential effect of SFN by decreasing the fibrotic markers collagen and α-SMA levels (Figure 2). While obstruction release partially decreases collagen deposition, as reported elsewhere [18,52], the combination of SFN and RUUO is more effective in halting disease progression (Figure 2). Notably, histological findings in the SFN + RUUO group did not show the SFN effect, possibly requiring more time for antioxidant administration to observe overall tissue recovery. Thus, another limitation includes the short time of SFN administration. Accordingly, our study determined SFN’s effects only up to seven days. Therefore, the total reduction in fibrosis could be observed with a long-term administration of SFN, which deserves future investigation. However, under our conditions, fibrotic markers were reduced, suggesting that SFN had beneficial effects at seven days, representing a preliminary study in which our antioxidant was beneficial in the short term.

SFN was capable of preventing mitochondrial damage, characterized by reduced mitochondrial biogenesis, which was prevented by SFN pretreatment. The enhancement of PGC-1α, NRF1, and PPAR-α induced by SFN (Figure 3A–D) indicates the induction of mitochondrial biogenesis, possibly related to Nrf2 activation followed by NRF1 induction because NRF1 promoter contains four Nrf2 binding sites [53]. However, if SFN-induced Nrf2 activation in the RUUO model is related to the enhancement of NFR1, it needs to be investigated. Moreover, SFN-induced Nrf2 activation has been linked to improvement in mitochondrial metabolism. A multiomics study in lung cells treated with SFN demonstrated that Nrf2 governs the expression of genes encoding protein members of respiratory complexes subunits, ATP synthase, and FA metabolism, suggesting that Nrf2 activation by SFN has mitochondrial biogenesis effects that go beyond the antioxidant effects of Nrf2 [54]. Additionally, it has been described that SFN affects the upregulation of mitochondrial biogenesis regulators [47,48,55,56]. Specifically, it has been reported that Nrf2 overregulates PGC-1α and TFAM in other models [57]. Supporting this, our previous study demonstrated that SFN activated mitochondrial biogenesis by PGC-1α/NRF1 pathway in the UUO model, reducing renal fibrosis [12].

Notably, our study does not include a positive control because using a “gold standard” in the RUUO model is still under investigation. For instance, previous findings in the RUUO model have reported the use of RAS blockers, such as losartan [24], aliskiren [46], non-steroid anti-inflammation drugs (diclofenac) [43], and xanthine oxidase inhibitors [51], which help in the recovery of renal function. Although these treatments have not been thoroughly investigated in the context of oxidative stress and mitochondrial function in the RUUO model, RAS inhibitors have been shown to protect mitochondria, promoting their restoration in other models. For instance, in diabetic mice, Losartan treatment reduced ROS, restoring mitochondrial morphology, dynamics, mitophagy and autophagy flux, ATP production, and preventing apoptosis [58]. In another study using diabetic mice, losartan promoted PGC-1α activation, preventing lipid accumulation [59]. An in vitro study in HK-2 cells showed that aliskiren restored autophagy [60]. Although a consistent and predictable effect of these treatments on mitochondria has not yet been demonstrated in RUUO, it would be an indispensable requirement for using positive control in our study.

In our study, SFN-induced biogenesis also increased VDAC (Figure 3E,F) and OXPHOS protein levels (Figure 4), restoring mitochondrial mass. Maintaining OXPHOS subunit levels also improve their activity and is associated with improved bioenergetics (Figure 4). Although CIII activity was unaffected in UUO and RUUO groups, mirroring previous UUO observations [12], its increase with SFN in RUUO suggests a boosting effect (Figure 4). Additionally, UQCRC2 is a direct target of NRF1 and is also transcriptionally activated by the adenosine monophosphate kinase (AMPK)/Nrf2 pathway, a signaling pathway regulated by SFN [61]. Thus, the UQCRC2 induction might be the result of SFN-induced NRF1 activation.

SFN further increases CPT1A levels (Figure 3E,G), a β-oxidation inductor regulated by PGC-1α/NRF1 at the transcriptional level [27]. FAs are crucial for ATP production in proximal tubule cells [62], and CPT1A alterations impair β-oxidation [22,63,64,65]. This impairment in UUO is linked to fibrosis and lipid accumulation [66], which activates macrophages that additionally increase the expression of fibrosis markers [36,67,68]. In addition, CPT1A deficiency is associated with fibrosis in other models of kidney damage [65,69,70], suggesting that the alteration of enzymes involved in β-oxidation contributes to CKD development. In accordance, other enzymes participating in β-the oxidation process, like acyl-CoA dehydrogenase family member 10 and medium-chain acetyl dehydrogenase, have been found to decrease kidney diseases [71].

Interestingly, SFN increases oxygen consumption related to β-oxidation in the P state, which suggests improved ATP production (Figure 5). Notably, ΔΨm is unchanged for any states (Figure 6), indicating that the mitochondrial membrane potential is not affected either during UUO or after its release. Regarding UUO, ΔΨm is unaffected because mitochondria did not suffer depolarization when β-oxidation-linked substrates were used, as previously reported [14]. Thus, the alterations in β-oxidation might be related to mitochondrial biogenesis decrease and not depolarized mitochondria. The fact that ΔΨm is unaffected during UUO is consistent with our prior CI and CII-linked respiration findings [14]. Therefore, declines in ATP-linked respiration are likely due to reduced mitochondrial mass rather than uncoupling or leak. In this way, we hypothesized that the SFN effect is related to the improvement of mitochondrial biogenesis, which increases mitochondrial mass, as we observed through increased VDAC, CPT1A, and OXPHOS subunit proteins. Increased CPT1A and OXPHOS restore bioenergetics via mitochondrial respiration (β-oxidation), which avoids lipid accumulation by preventing fatty acid consumption.

A consequence of altered β-oxidation is the accumulation of FA in the cytosol in lipid droplet form, as seen in Oil Red-O staining (Figure S2). In addition, CD36 overexpression on the cell membrane further increases FA uptake [32], leading to lipid production, including TGs, which accumulate rapidly after UUO [72]. As we showed, the overexpression of CD36 continues even after the obstruction is removed, which is prevented with SFN. Notably, CD36 is linked to collagen-inducing gene expression, accelerating fibrosis [34]. Interestingly, TGs and lipid accumulation tend to decrease in the RUUO group (Figure 7 and Figure S2). Whether both parameters continue to decline with obstruction release alone over time should be determined in further studies. While we found no changes in DGAT1A levels, a slight tendency to increase was observed in UUO and RUUO groups (Figure 7D,E), and a decrease in SFN + RUUO group, which might suggest that SFN effectively reduces TGs in the RUUO model. This is supported by SFN-induced reduction in PPAR-γ in the RUUO group (Figure 7A,C), suggesting that persistent lipid accumulation and altered FA metabolism are prevented by this antioxidant (Figure 7).

Bioenergetic alterations and lipid accumulation cause mitochondrial dynamics disruption, leading to a shift of mitochondria toward fission [10,73,74]. Under these conditions, DRP1 is recruited to the OMM, resulting in mitochondrial fragmentation [38]. Our results showed elevated DRP1 levels in UUO that slightly decreased after RUUO, most notably with SFN (Figure 8A,B), suggesting SFN’s role in mitigating fission. A study reported that SFN can decrease fission through DRP1 levels [75], suggesting a direct effect of this antioxidant on DRP1. It is unknown if only SFN administration might directly act through this fission protein. Thus, future investigations might clarify the relevance of SFN in mitochondrial fission. Additionally, SFN increased MFN2 levels, indicating an improvement in mitochondrial fusion. Previous data using the UUO model suggest that SFN alone does not fully recover fusion in the obstructed kidneys [12], perhaps requiring obstruction removal for full effect.

Mitophagy is essential to clear damaged mitochondria, and excessive fission promotes the activation of mitophagy [76]. Previous studies showed that mitophagy and autophagy are altered in UUO [77,78]. Our results showed that PINK1 is lightly reduced in UUO and RUUO groups, but Parkin increased in the RUUO group without changes in UUO animals, suggesting a possible alteration of this mechanism (Figure 9A–C). The elevation in Parkin levels was reported after two weeks of removing the obstruction [52], which might be interpreted as a regulatory mechanism. However, to clarify our findings concerning mitophagy, we evaluated macroautophagy, better known as autophagy. Autophagy is a process that also degrades dysfunctional mitochondria [79]. In RUUO, increased Beclin levels indicated the induction of autophagy through the formation of the autophagosomes. Conversely, p62 increased while the ratio LC3-II/LC3-I decreased (Figure 9), related to autophagic body accumulation and the lack of lipidation of the autophagosome membrane, respectively [80,81]. Both data strongly suggest that the autophagic process is not being completed in the RUUO model. Although UUO animals did not show altered Beclin and p62 levels, the LC3-II-LC3-I ratio decreased, similar to previous reports [12] (Figure 9), highlighting the idea of impaired mitophagy flux. In accordance, electron microscopy micrographs showed the presence of autophagosomes in mitochondria from UUO rats. Concerning RUUO, empty vacuoles were observed (Figure 9J), indicating that the autophagic process was stopped after the release of UUO. The impairment of autophagy in RUUO animals might result from oxidative stress generated during RUUO surgery [82], potentially caused by lipid accumulation and metabolic disturbance leading to reactive oxygen species (ROS) overproduction and lipoperoxidation [83]. These peroxidation products, including malondialdehyde and 4-hydroxynonenal, persist after obstruction [82], suggesting that the effect of SFN on mitophagy may be related to its antioxidant properties.

**Figure 10:**
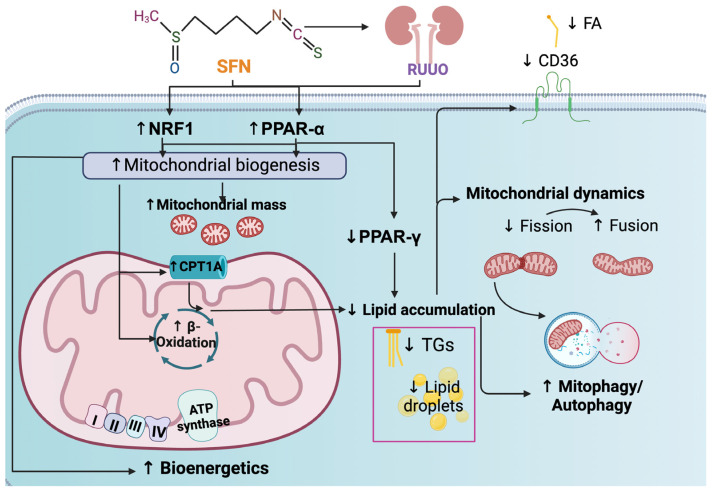
Integrative scheme. Pre-administration of sulforaphane (SFN) in releasing unilateral ureteral obstruction (RUUO) activates nuclear respiratory factor 1 (NRF1), peroxisome proliferator-activated receptor-α (PPAR-α), leading to mitochondrial biogenesis. The latter increases mitochondrial mass and carnitine palmitoyl transferase 1A (CPT1A), enhancing β-oxidation and favoring the bioenergetics process. Promoting β-oxidation prevents lipid accumulation, which decreases lipid droplets and triglycerides (TGs) synthesis. Additionally, peroxisome proliferator-activated receptor-γ (PPAR-γ) and the cluster of differentiation 36 (CD36) levels, as well as the uptake of fatty acids (FA), decrease, further reducing lipid accumulation. The reduction in lipid accumulation positively restores mitochondrial dynamics by decreasing fission and increasing fusion and the mitophagy/autophagy flux. ↑: increase; ↓: decrease; oxidative phosphorylation (OXPHOS) proteins: mitochondrial complexes I-IV (abbreviated as I, II, III, and IV) and adenosine triphosphate (ATP) synthase.

In summary, SFN alleviates mitochondrial impairment after RUUO by enhancing mitochondrial biogenesis, restoring β-oxidation, and thus reversing lipid accumulation, preventing fission, and altering mitophagy defects. Nrf2 activation likely contributes to biogenesis enhancement and fibrosis reduction, avoiding further damage (Figure 10). The limitations of our study are associated with the use of one dose of SFN, the short study duration, and the lack of positive control. Therefore, it might be valuable to include future long-term studies with additional doses of SFN and employ a positive control to investigate if its effects are dose-dependent, increased, or canceled. The latter could be of clinical relevance because, to our knowledge, no antioxidant therapy has been utilized in the UUO release surgery.

The findings of this study encourage future research on SFN as a potential mitochondria-targeted therapy for ON, which could be intended in clinical approaches with significant implications for developing novel treatments for ON in adults and children. In addition, the RUUO rat model simulates the reestablishment of urinary flow after UUO, allowing for a conductive study of renal damage and its repair after removing the obstruction. This model is closer to what happens in the clinical scenario, enabling treatments that may be utilized in future clinical research. After the release of the obstruction in patients, treatments are focused on the recovery of renal function; however, little is addressed as to the complete restoration of other parameters involving mitochondrial function. In this sense, some of the patients undergoing UUO release surgery develop CKD. Thus, continuing with pre-clinical and clinical studies using SFN in obstructive nephropathy could lead to the development of better treatments in the future of this pathology. Human clinical models have shown the beneficial effects of SFN for prostate cancer (a phase II study) [84,85], cardiovascular diseases [86], and type 2 Diabetes mellitus (T2DM) [87], highlighting the importance of following the studies of this antioxidant in a nephropathy-obstructive context. In our research, SFN emerges as a promising therapeutic agent in ON-induced CKD along with obstruction release, which could potentiate the recovery of renal functions using a more targeted approach. This study again put mitochondria as a center of damage in UUO and RUUO, which could open new alternatives in the study of SFN, focusing on this organelle at a clinical level.

## 5. Conclusions

Our findings demonstrate that the effect of SFN in the RUUO model is mediated by the enhancement of mitochondrial functions such as mitochondrial biogenesis, β-oxidation, dynamics, and mitophagy. These processes, in turn, lead to increased mitochondrial mass and improved ETS activities in the kidney, mitigating lipid accumulation and associated mitochondrial dysfunction, such as excessive fission and impaired mitophagy/autophagy. SFN ultimately prevents fibrosis progression in this model by addressing these critical pathophysiological mechanisms. Elucidating the processes underlying kidney tissue and functional recovery after obstruction release has significant implications for developing novel therapies for ON in adults and children.

## Data Availability

The original contributions presented in this study are included in the article/Supplementary Material. Further inquiries can be directed to the corresponding author.

## Supplementary Materials

The following supporting information can be downloaded at https://www.mdpi.com/article/10.3390/antiox14030288/s1, Figure S1: Representative micrographs of renal damage of the kidney cortex from the different experimental groups; Figure S2: Sulforaphane (SFN) decreases lipid deposition in the release of unilateral ureteral obstruction (RUUO) model. Table S1: List of antibodies used for immunoblots.

## Abbreviations

α-SMA	alpha-smooth muscle actin
ADP	adenosine diphosphate
AMPK	adenosine monophosphate kinase
Ant A	antimycin A
ATP	adenosine triphosphate
ATP5A	ATP synthase-α subunit
CCCP	carbonyl cyanide m-chlorophenylhydrazone
CD36	cluster of differentiation 36
Col IV	collagen IV
CI	complex I
CII	complex II
CIII	complex III
CIV	complex IV
CPT1A	carnitine palmitoyl transferase 1A
CKD	chronic kidney disease
cyt c	cytochrome c
DCPIP	dichlorophenolindophenol sodium salt hydrate
DGAT1	diacylglycerol acyl transferase 1
DRP1	dynamin-related protein 1
DUB	decyl-ubiquinone
DUBH_2_	reduced decyl-ubiquinone
EDTA	ethylenediaminetetraacetic acid
EGTA	ethylene glycol-bis(2-aminoethyl ether)-N,N,N’,N’-tetraacetic acid
ETS	electron transfer system
FA	fatty acids
FAF-BSA	fatty acid-free bovine serum albumin
FN	fibronectin
GAPDH	glyceraldehyde 3-phosphate dehydrogenase
H&E	hematoxylin-eosin
HEPES	4-(2-hydroxyethyl)-1-piperazine ethane sulfonic acid
kDa	kilodaltons
KOH	potassium hydroxide
LC3-I/LC3-II	microtubule-associated proteins 1A/1B light chain 3
ΔΨm	mitochondrial membrane potential
MAL	malate
MFN1	mitofusin 1
MFN2	mitofusin 2
MTCO1	mitochondrially encoded cytochrome c oxidase 1
mtDNA	mitochondrial deoxyribonucleic acid
NAD^+^	oxidized nicotinamide adenine dinucleotide
NADH	reduced nicotinamide adenine dinucleotide
NADP^+^	oxidized nicotinamide adenine dinucleotide phosphate
NADPH	reduced nicotinamide adenine dinucleotide phosphate
NaF	sodium fluoride
NaH_2_PO_4_	sodium phosphate monobasic
Na_2_HPO_4_	sodium phosphate dibasic
Na_3_VO_4_	sodium orthovanadate
NDUFB8	NADH:ubiquinone oxidoreductase subunit B8
NRF1	nuclear respiratory factor 1
Nrf2	nuclear factor erythroid 2-related factor 2
OMM	outer mitochondrial membrane
ON	obstructive nephropathy
OXPHOS	oxidative phosphorylation
P	OXPHOS-associated respiration
p62	sequestosome 1
PAGE	polyacrylamide gel electrophoresis
PBS	phosphate buffer saline
PGC-1α	peroxisome proliferator-activated receptor γ co-activator-1α
PINK1	phosphatase and tensin homolog deleted on chromosome 10 (PTEN)-induced kinase 1
PPAR-α	peroxisome proliferator-activated receptor-α
PPAR-γ	peroxisome proliferator-activated receptor-γ
RAS	renin-angiotensin system
RC	respiratory control
RIPA	radioimmunoprecipitation assay
ROS	reactive oxygen species
Rot	rotenone
RUUO	release of unilateral ureteral obstruction
SFN + RUUO	RUUO treated with sulforaphane
S	state
Saf	safranin O
SDHB	succinate dehydrogenase complex iron-sulfur subunit B
SDS	sodium dodecyl sulfate
SEM	standard error of the mean
SFN	sulforaphane
TEM	transmission electron microscopy
TFAM	mitochondrial transcription factor A
TGs	triglycerides
TNB	5-thio-2-nitrobenzoic acid
Tris	Trizma base
UUO	unilateral ureteral obstruction
UQCRC	ubiquinol-cytochrome c reductase core protein 2
VDAC	voltage-dependent anion channel
