# Supplementary material for: Sulforaphane Restores Mitochondrial β-Oxidation and Reduces Renal Lipid Accumulation in a Model of Releasing Unilateral Ureteral Obstruction
Source: Antioxidants (Basel). 2025 Feb 28;14(3):288. doi: 10.3390/antiox14030288 (PMC11939561; doi:10.3390/antiox14030288)
Supplement: Supplementary file 1 [file antioxidants-14-00288-s001.zip › antioxidants-3459916-supplementary.pdf]

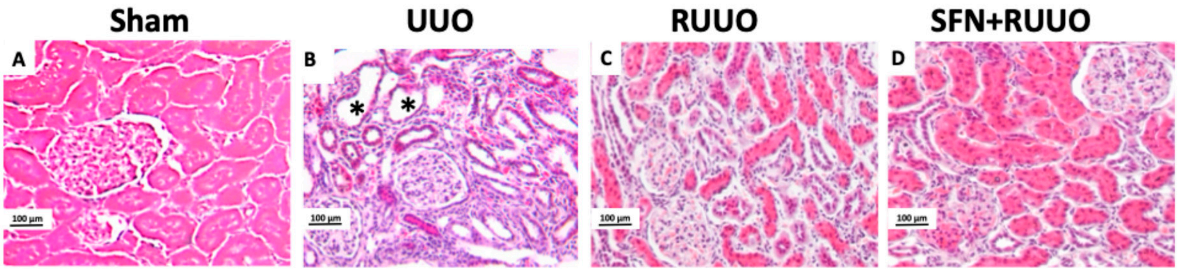

Figure S1. Representative micrographs of renal damage of the kidney cortex from the different experimental groups. A) Sham group shows normal kidney histology. B) Unilateral ureteral obstruction (UUO) group shows numerous proximal convoluted tubules revested by flattened atrophic epithelium (black asterisks). C) Releasing unilateral ureteral obstruction (RUUO) rats showed lesser tubular damage. D) RUUO treated with sulforaphane (SFN) (RUUO+SFN) shows even lesser damaged proximal convoluted tubules. n=3 per group.

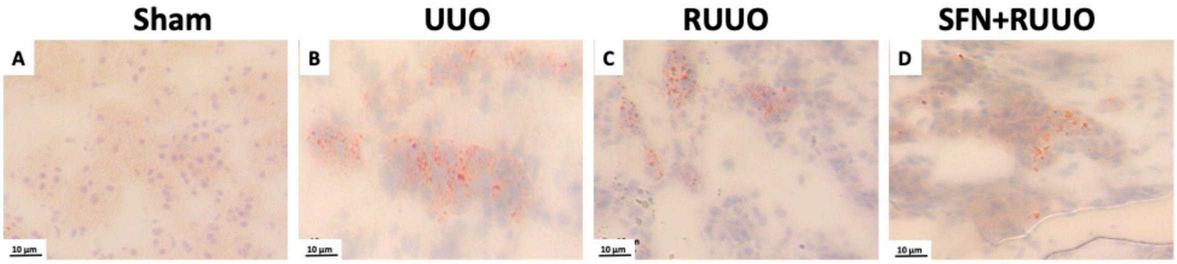

Figure S2. Sulforaphane (SFN) decreases lipid deposition in the release of unilateral ureteral obstruction (RUUO) model. Red oil staining does not show lipids in the kidney section from A) sham rat. B) Many red dots in the cytoplasm of cortical tubular epithelium in the kidney of UUO rats correspond to lipidic vacuoles. C) A smaller amount of lipid vacuoles is seen in the cytoplasm of tubular epithelial cells from RUUO rats. D) Occasional cytoplasmic lipid vacuoles in the tubular epithelium are observed in the SFN+RUUO group.

Supplementary Table S1. List of antibodies used for immunoblots.

Antibody	Full name	Source	Catalog number	Manufacturer	Dilution
Anti-FN	Fibronectin	Mouse	sc-271098 mouse	Santa Cruz Biotechnology	1:2000
Anti-α-SMA	Alpha-smooth muscle actin	Rabbit	GTX10034	GeneTex	1:2000
Anti-Col IV	Collagen IV	Mouse	SAB4200500	Sigma Aldrich	1:3000
Anti-PGC-1α	Peroxisome proliferator-activated receptor-gamma coactivator (PGC)-1alpha	Rabbit	AB3242	Sigma Aldrich	1:2000
Anti-NRF1	Nuclear respiratory factor 1	Rabbit	46743	Cell Signaling Technology	1:2000
Anti-VDAC	Voltage-dependent anion channel	Rabbit	V2139	Sigma Aldrich	1:2000
Anti-TFAM	Transcription factor A mitochondrial	Rabbit	7495S	Cell Signaling Technology	1:1000
Anti-OXPHOS	Total oxidative phosphorylation (OXPHOS) rodent western blot (WB) antibody cocktail	Mouse	ab110413	Abcam	1:10000
Anti-DRP1	Dynamin related protein 1	Rabbit	sc-32898	Santa Cruz Biotechnology	1:2000
Anti-MFN1	Mitofusin 1	Rabbit	sc-50330 rabbit	Santa Cruz Biotechnology	1:2000
Anti-MFN2	Mitofusin 2	Rabbit	9482S	Cell Signaling Technology	1:2000
Anti-PINK1	PTEN-induced kinase 1	Rabbit	Ab23707	Abcam	1:3000
Anti-Parkin	Parkin	Rabbit	Ab15954	Abcam	1:2000
Anti-beclin	Beclin	Mouse	MAB5295	R&D Systems	1:3000

Supplementary Table S1. List of antibodies used for immunoblots (Cont.).

Antibody	Full name	Source	Catalog number	Manufacturer	Dilution
Anti-p62	Sequestosome	Rabbit	P0067	Sigma Aldrich	1:3000
Anti-LC3	Microtubule-associated proteins 1A/1B light chain 3	Rabbit	L7543	Sigma Aldrich	1:3000
Anti-CD36	Cluster of differentiation 36	Rabbit	GTX55559	GeneTex	1:1000
Anti-PPAR-α	Peroxisome proliferator-activated receptor-alpha	Rabbit	Ab24509	Abcam	1:1000

Anti-PPAR-γ	Peroxisome proliferator-activated receptor-gamma	Rabbit	PA3-821A	Thermo Fisher Scientific	1:1000
Anti-CPT1A	Carnitine palmitoyl transferase I	Rabbit	Ab234111	Abcam	1:2000
Anti-DGAT1	Diacylglycerol O-acyltransferase 1	Rabbit	GTX48577	GeneTex	
Anti-GAPDH	Glyceraldehyde 3-phosphate dehydrogenase	Mouse	Ab8245	Abcam	1:5000

Abcam Limited. Waltham, MA, USA.
Cell Signaling Technology Inc. Danvers, MA, USA.
GeneTex Inc. Irvine, CA, USA.
R&D Systems Inc. Minneapolis, MN, USA.
Santa Cruz Biotechnology Inc. Dallas, TX, USA.
Sigma Aldrich Inc. St. Louis, MO, USA.
Thermo Fisher Scientific Inc. Waltham, MA, USA.
